# Supplementary material for: Sources of confidence in value-based choice
Source: Nat Commun. 2021 Dec 17;12:7337. doi: 10.1038/s41467-021-27618-5 (PMC8683513; doi:10.1038/s41467-021-27618-5)
Supplement: Supplementary file 1 — Supplementary Information [file 41467_2021_27618_MOESM1_ESM.pdf]

Sources of confidence in value-based choice Supplementary figures

Jeroen Brus[1,2,✉], Helena Aebersold[3], Marcus Grueschow[4], and Rafael Polania[1,2,✉]

[1]Decision Neuroscience Lab, Department of Health Sciences and Technology, ETH Zurich
[2]Neuroscience Center Zurich, Switzerland
[3]Epidemiology, Biostatistics and Prevention Institute, University of Zurich, Zurich, Switzerland
[4]Zurich Center for Neuroeconomics (ZNE), Department of Economics, University of Zurich, Zurich, Switzerland

Supplementary Fig. 1

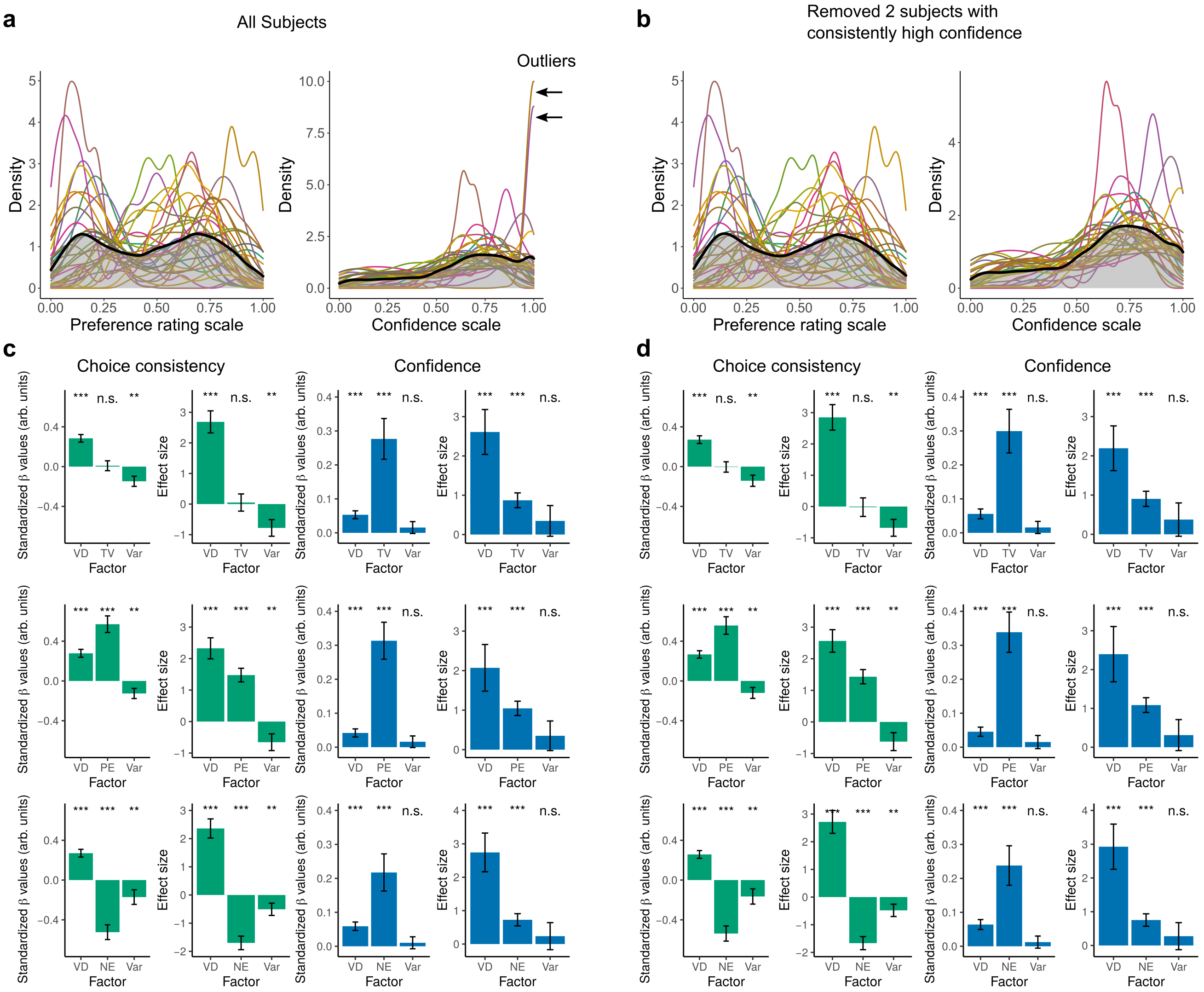

Usage of the preference and confidence rating scale and a comparison of the regression analysis with and without outliers. **a)** Density plot of the usage of the preference and confidence rating scale. Colored lines correspond to individual participants, black lines and gray shadings are aggregated data over all subjects. Participants use the full preference rating scale and mostly the positive part of the confidence rating scale. There are two outlier participants consistently reporting high confidence, indicated by the two arrows. **b)** Usage of the preference and confidence rating scale with the two outliers removed. **c&d)** Multiple regression analyses of choice consistency and confidence. Total value, positive evidence and negative evidence are used as independent variables in different regressions, while controlling for value difference and variability. Data from all subjects are used in c and the outliers were removed in d. We find no major differences when we exclude the two participants. Error bars indicate the mean standard deviation of the posterior estimates in the hierarchical mixed-effects models. Stars indicate P-values as follows: * $P < 0.05$, ** $P < 0.01$ and *** $P < 0.001$, n.s. indicates a P-value > 0.05. P-values are based on the highest density interval of the posterior estimates. For the left part of the figure n = 33 independent participants, for the right n = 31.

Supplementary Fig. 2

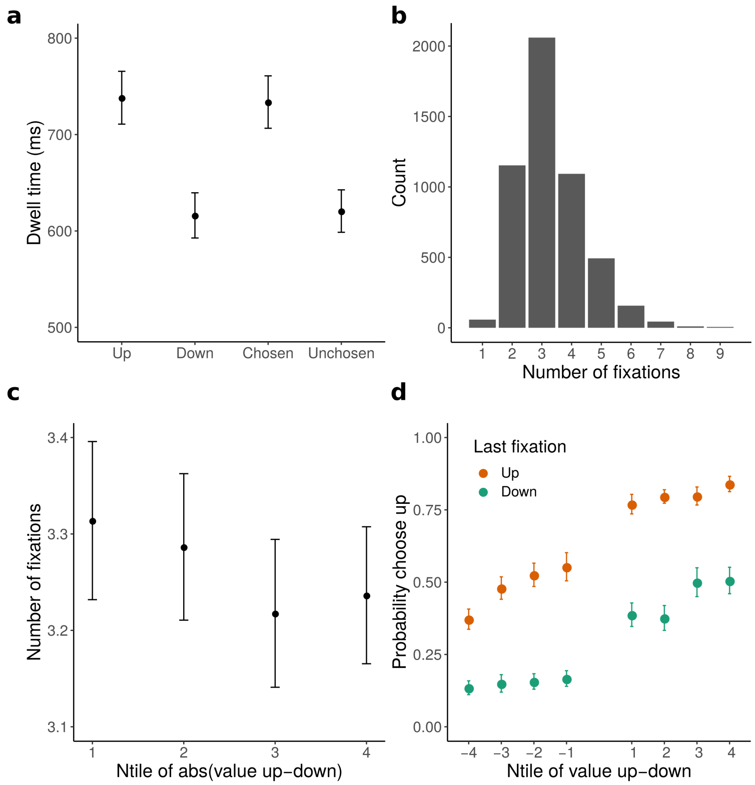

Eye tracking data. a) Mean dwell times on the item presented up, down and of the chosen and unchosen item. Participants look longer at the item they choose and tend to look longer at the item presented at the top. In panel a, c & d data are presented as mean values $\pm$ SEM. **b)** Participants switch between looking at the upper and lower item. The number of fixations ranges between 1 and 9, all trials in which participants only fixated on one item were removed. This way 1.1% of trials were removed. **c)** Number of fixations relates to the absolute of the difference in value between the upper item and the lower item. **d)** Psychometric choice curve conditional on the location of the last fixation. While participants choose, they tend to look more often at the item of choice. For the whole figure n = 33 independent participants.

Supplementary Fig. 3

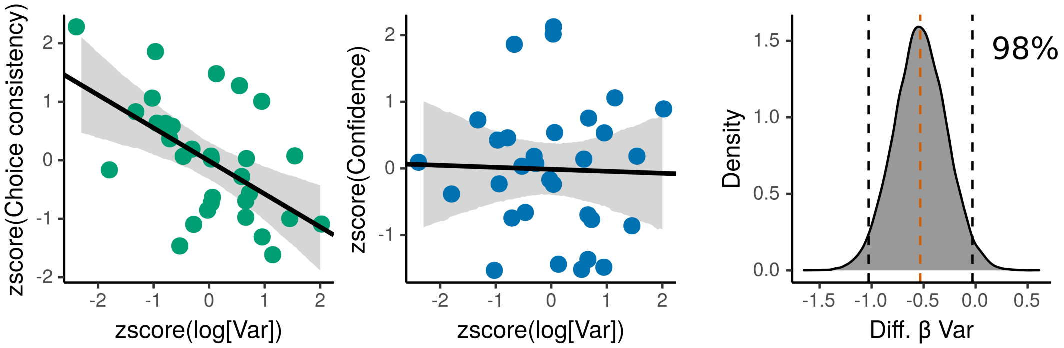

Effects of trial-to-trial variability on choice consistency and confidence at the participant level. This is a replication of Figure 1 d, however two outlier subjects who consistently report high confidence have been excluded (see Supplementary Figure 1). Participant's average level of variability in the rating task had a negative influence on average choice consistency of that participant ($\beta = -0.56 \pm 0.17$, $P < 0.001$, $r = -0.55$), however this effect is not present for the same analyses performed on confidence reports ($\beta = -0.03 \pm 0.19$, $P = 0.44$, $r = -0.03$). Gray shaded areas indicate 95% confidence bands. The difference of the effect of average variability on choice consistency and confidence ratings is significant since 98% of the density of the posterior estimates is below zero ($\Delta\beta_{cons-conf} = -0.53 \pm 0.25$, $P = 0.02$). Vertical black dashed lines indicate the 95 % confidence interval. For the whole figure n = 31 independent participants.

Supplementary Fig. 4

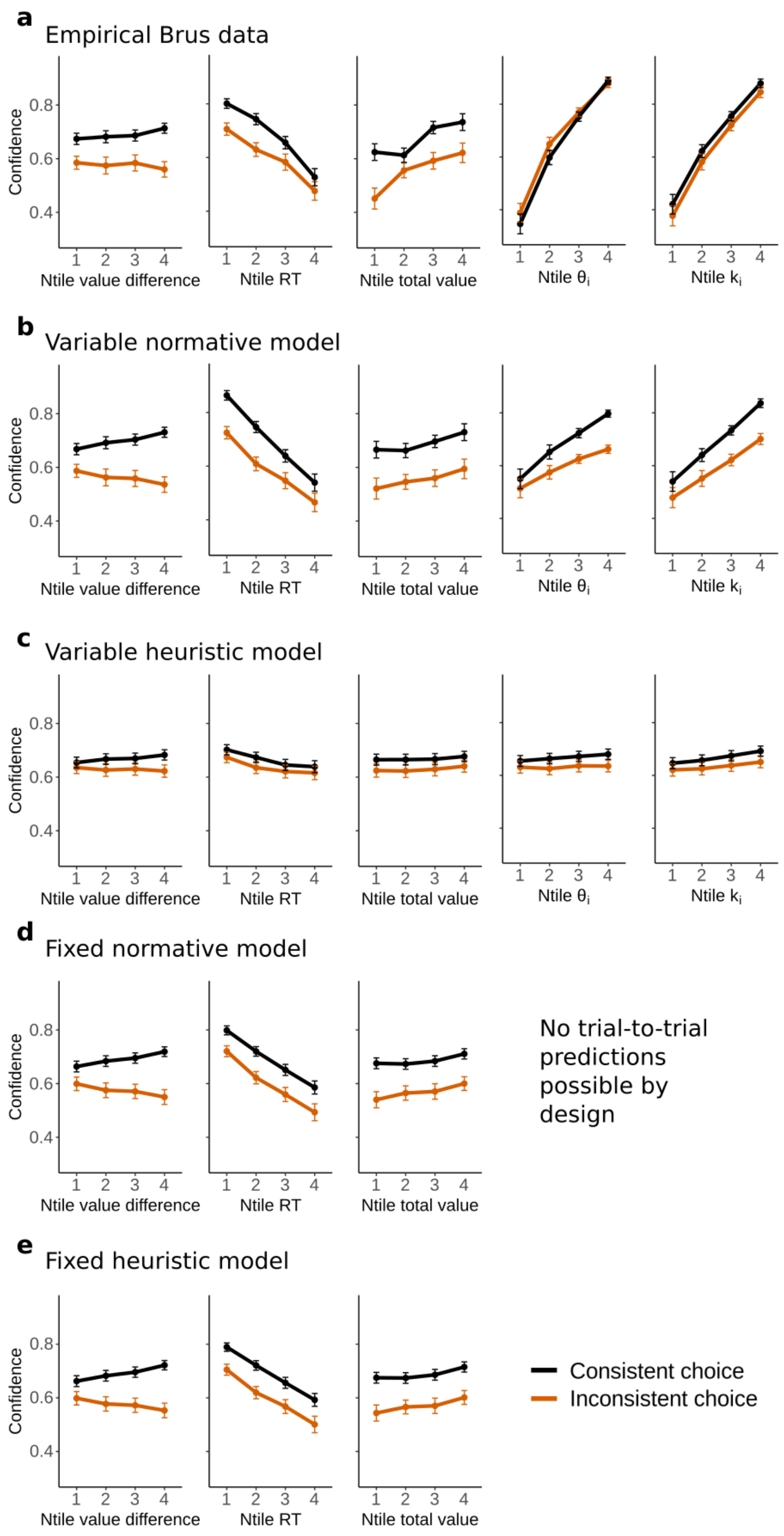

Confidence predictions. a) Empirical confidence data. **b-e)** Confidence predictions of the b) variable normative, c) variable heuristic, d) fixed normative and e) fixed heuristic models with respect to the value difference, reaction time, total value, estimated θ_i and k_i value. For the fixed models there are no estimated θ_i and k_i values by design. Points represent mean values and error bars the s.e.m. Predictions are based on the Brus data. For the whole figure n = 33 independent participants.

Supplementary Fig. 5

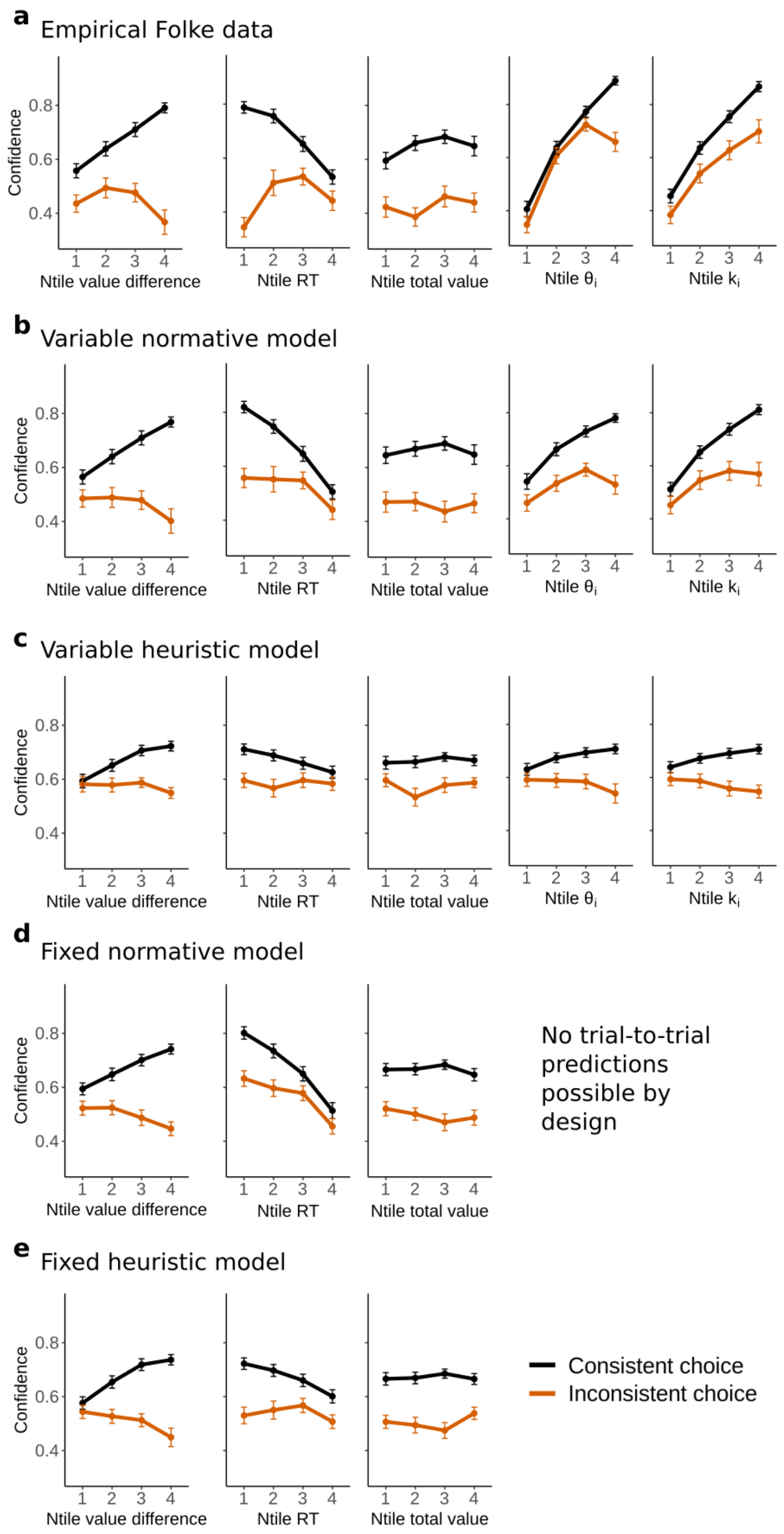

Confidence predictions. Same as Supplementary Fig. 4, but for the Folke data (1), n = 28 independent participants.

Supplementary Fig. 6

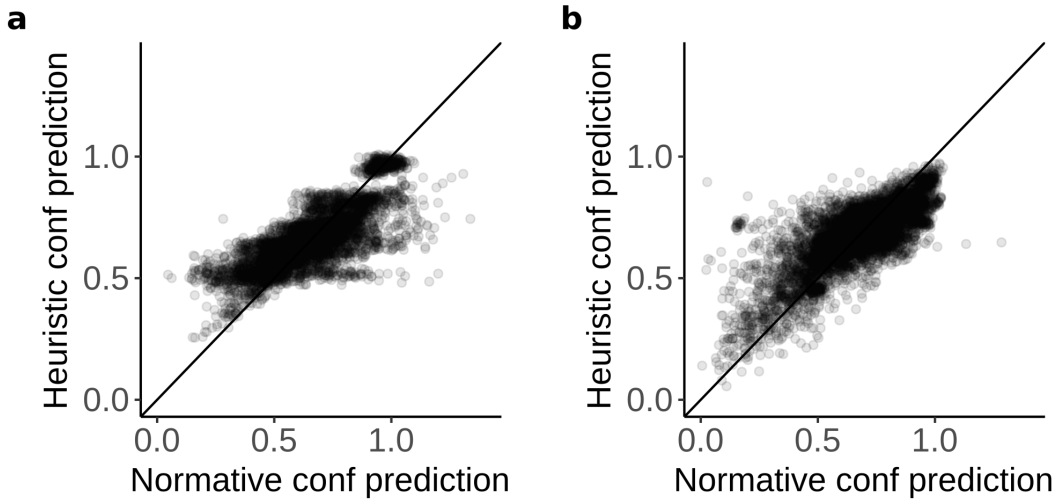

Normative vs. heuristic confidence predictions. a) Confidence predictions of the variable normative model plotted versus the confidence predictions of the variable heuristic model. Diagonal line represents equality. Predictions are based on the Brus data. **b)** Same as a, but based on the Folke data (1).

Supplementary Fig. 7

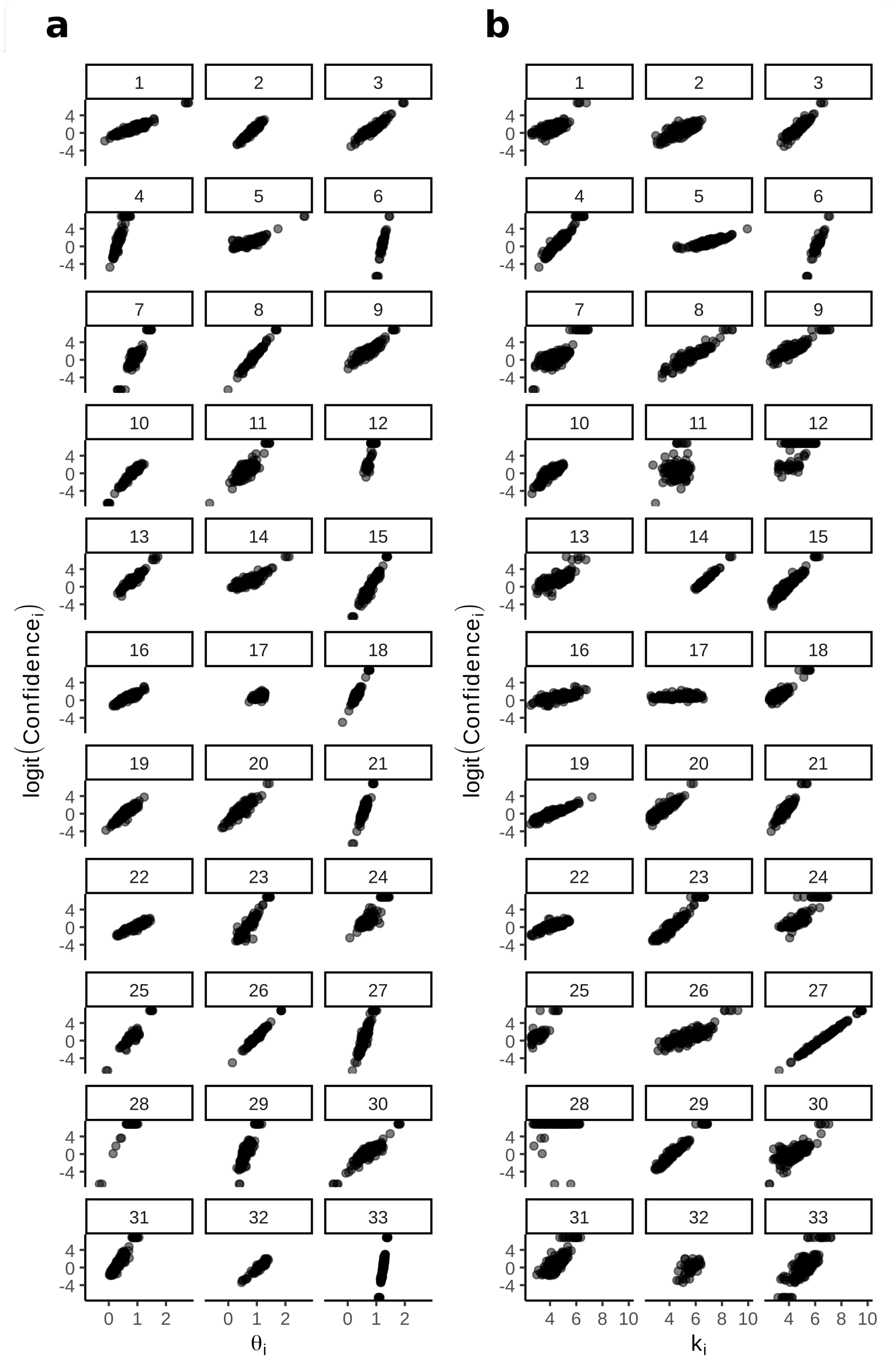

Positive relation between confidence and θ_i and k_i. With the joint modelling approach we inferred values of θ_i and k_i for all trials. For most subjects there exists a strong positive relation between θ_i, k_i and confidence. **a)** For each individual subject the confidence on each trial is plotted versus the estimated θ_i. **b)** Same as a) but for the relation of confidence and the estimated k_i. Based on the Brus data.

Supplementary Fig. 8

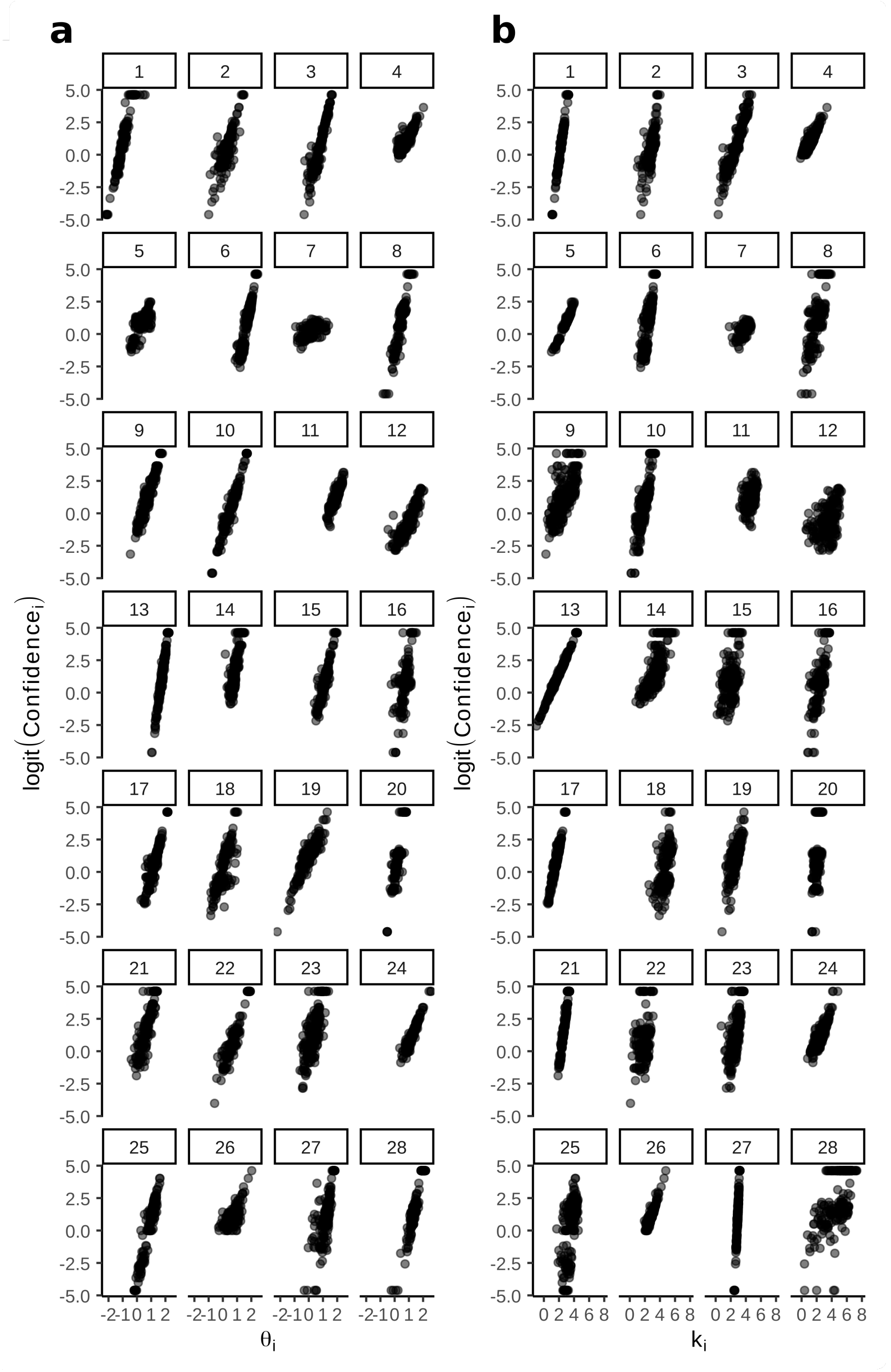

Relation between confidence and θ_i and k_i. Same as Supplementary Fig. 7, but for the Folke data (1).

Supplementary Fig. 9

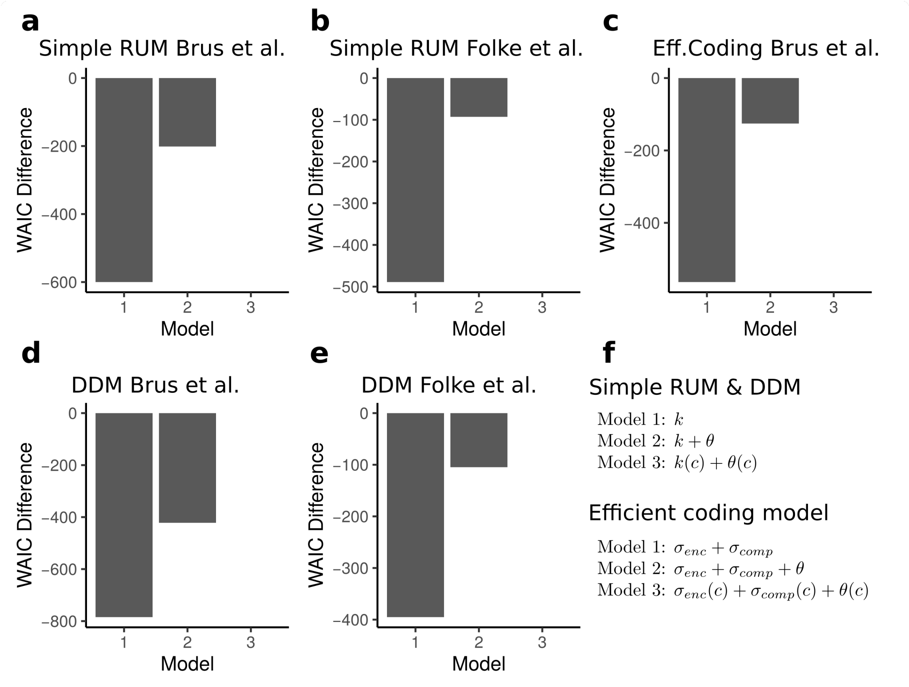

Model comparison. a-e) WAIC model comparison of three alternatives of the RUM, efficient coding model and DDM based on our own data and the dataset of Folke et al. (1) **f)** Indication of the difference between the models. RUM & DDM: Model 1 is the most basic model in which only the evidence gain is included, in Model 2 the attentional discount factor is included and in Model 3 both the evidence gain as well as the attentional discount factor are allowed to vary with confidence. Efficient coding model: Model 1 includes parameters representing encoding noise and comparison noise, in Model 2 the attentional discount factor is added and in Model 3 these parameters are allowed to vary with confidence.

Supplementary Fig. 10

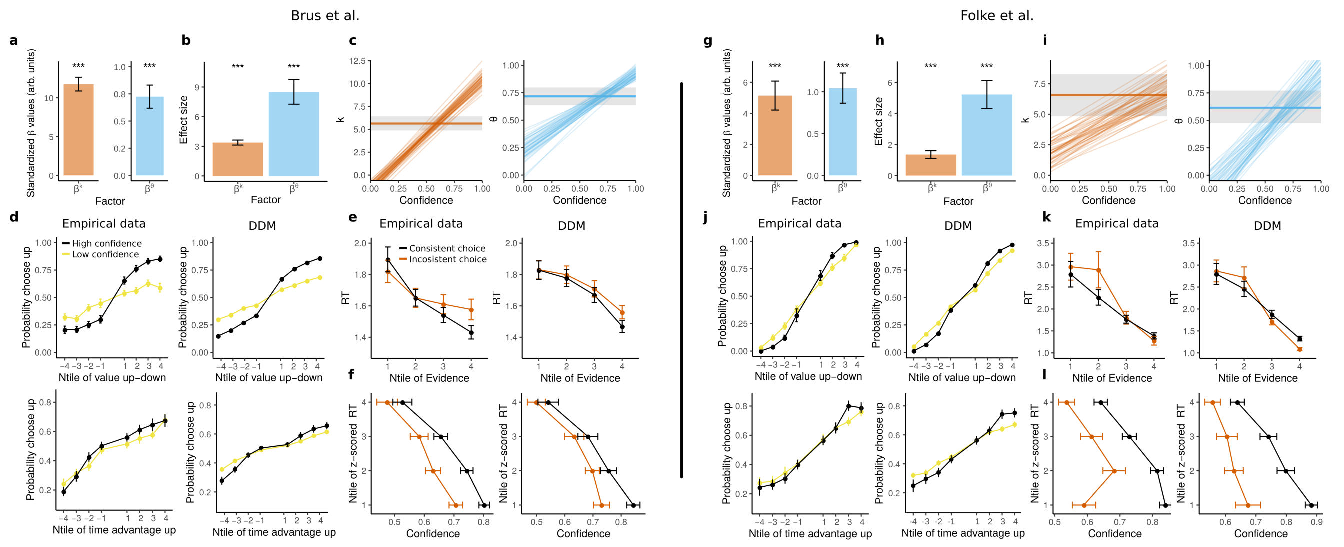

Experimental data and DDM modeling results. **a)** Standardized posterior estimates of the beta values of the relation between confidence and k and θ. Error bars indicate the mean posterior estimate of the standard deviation. Both β^k and β^θ are significantly bigger than zero with P<0.001. For the whole figure P-values are based on the highest density interval of the posterior estimates. **b)** Effect sizes of the results shown in a). Error bars indicate the standard deviation of the posterior estimates of the mean of the effect size. Both the effect sizes of β^k and β^θ are significantly bigger than zero with P<0.001. **c)** Comparison of parameter estimates of two alternative aDDMs: an aDDM with agent-specific estimates of k and θ and a RUM that allows for trial-to-trial fluctuations of k and θ. Left: the median of the posterior estimate of k of the agent-specific RUM is indicated as the horizontal orange line, the shaded grey area indicates the 95% confidence interval. The diagonal orange lines represent 100 random samples of the posterior distribution of how k changes with confidence in the aDDM allowing for trial-to-trial fluctuations. Right: the same as left, but for θ. **d)** Left column: the empirical probabilities of choosing the upper item; up: as a function of value difference; down: as a function of the difference in dwell time. Right column: the same as left but for the predicted probabilities of choosing the upper item by the aDDM. The trials are median split in high/low confidence and color coded in respectively black/yellow. Value difference and dwell time difference are split into eight groups of equal size. In panel d, e, j & k data are presented as mean values $\pm$ SEM. **e)** Left: empirical reaction times as a function of the evidence (absolute value difference) split in consistent and inconsistent choices. Right: predictions of the aDDM. **f)** Left: empirical reaction times as a function of confidence split in consistent and inconsistent choices. Right: predictions of the aDDM. **g-k)** Same as a-f, but for the data generated by Folke et al. (1). For the Brus et al. dataset n = 33 independent participants, for Folke et al. n = 28 independent participants.

Supplementary Fig. 11

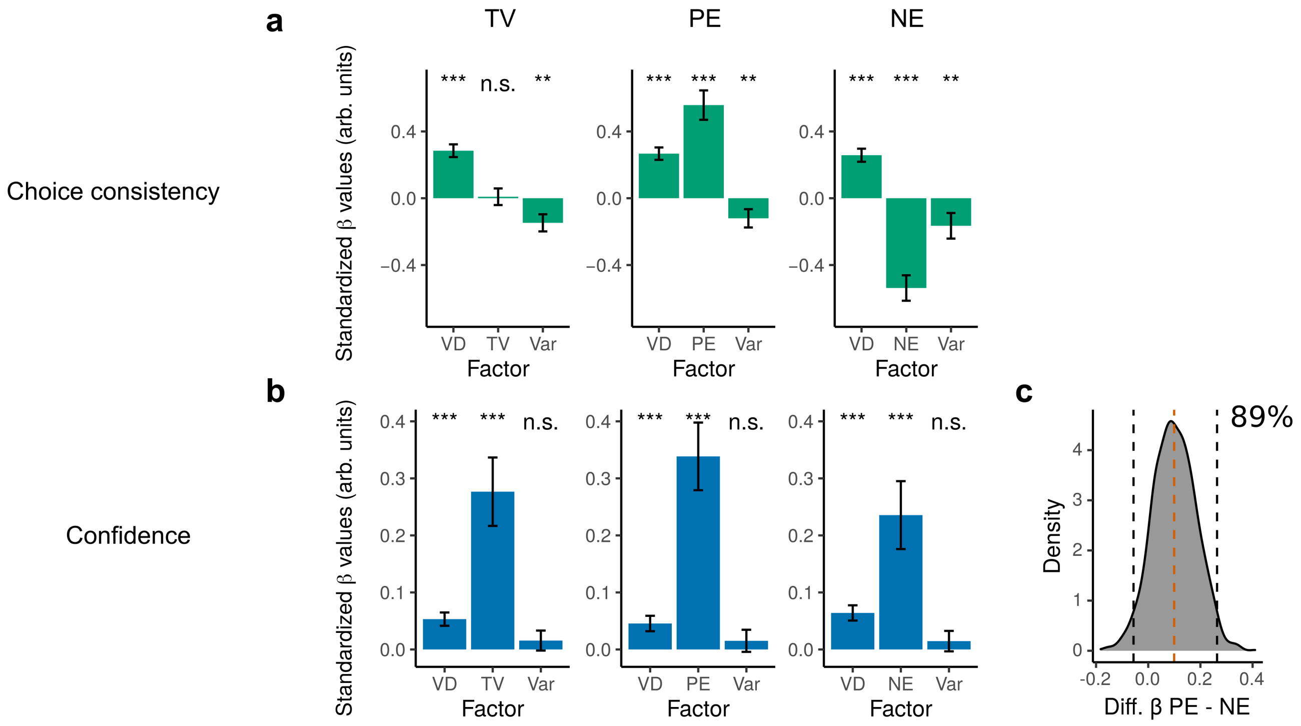

Multiple regression analyses of choice consistency and confidence, comparing the effect of total value (TV), positive evidence (PE) and negative evidence (NE). a&b) Standardized estimates of three multiple regression analyses of choice consistency and confidence. Total value, positive evidence and negative evidence are used as explanatory variables in separate regressions, while controlling for value difference and variability. Note that in a single regression model, value difference (VD), total value (TV), positive evidence (PE) and negative evidence (NE) cannot be used as factors as there will be collinearities. Supporting the results presented in Figure 1 of the main article, we found that the effects of variability are indeed present in all choice models and absent in all confidence models. Furthermore, PE and NE both influence choice accuracy, but PE affects confidence more strongly. However, based on the experimental procedure adopted in our study, we cannot ascertain whether the true factor underlying this phenomenon is TV or PE as by design these two factors are highly correlated. Error bars indicate the mean standard deviation of the posterior estimates in the hierarchical mixed-effects models. Stars indicate P-values as follows: * $P < 0.05$, ** $P < 0.01$ and *** $P < 0.001$, n.s. indicates a P-value > 0.05. P-values are based on the highest density interval of the posterior estimates. **c)** Investigating the difference of the beta values of PE and NE on confidence we find that there is a trend for the effect of PE to be larger, however this effect is not significant (11% of the density is below 0). Vertical red dashed line indicates the median, black dashed lines indicate the 95 % confidence interval. For the whole figure n = 33 independent participants.

Supplementary References

1. Tomas Folke, Catrine Jacobsen, Stephen M. Fleming, and Benedetto De Martino. Explicit representation of confidence informs future value-based decisions. *Nature Human Behaviour*, 1(1):17–19, 2017. ISSN 23973374. doi: 10.1038/s41562-016-0002.
