# Supplementary material for: Sources of confidence in value-based choice
Source: Nat Commun. 2021 Dec 17;12:7337. doi: 10.1038/s41467-021-27618-5 (PMC8683513; doi:10.1038/s41467-021-27618-5)
Supplement: Supplementary file 3 — Reporting summary [file 41467_2021_27618_MOESM3_ESM.pdf]

nature portfolio

Corresponding author(s): Jeroen Brus and Rafael Polania

Last updated by author(s): Nov 18, 2021

Reporting Summary

Nature Portfolio wishes to improve the reproducibility of the work that we publish. This form provides structure for consistency and transparency in reporting. For further information on Nature Portfolio policies, see our Editorial Policies and the Editorial Policy Checklist.

Statistics

For all statistical analyses, confirm that the following items are present in the figure legend, table legend, main text, or Methods section.

n/a	Confirmed	
☐	☒	The exact sample size (*n*) for each experimental group/condition, given as a discrete number and unit of measurement
☐	☒	A statement on whether measurements were taken from distinct samples or whether the same sample was measured repeatedly
☐	☒	The statistical test(s) used AND whether they are one- or two-sided<br>*Only common tests should be described solely by name; describe more complex techniques in the Methods section.*
☒	☐	A description of all covariates tested
☐	☒	A description of any assumptions or corrections, such as tests of normality and adjustment for multiple comparisons
☐	☒	A full description of the statistical parameters including central tendency (e.g. means) or other basic estimates (e.g. regression coefficient) AND variation (e.g. standard deviation) or associated estimates of uncertainty (e.g. confidence intervals)
☐	☒	For null hypothesis testing, the test statistic (e.g. *F*, *t*, *r*) with confidence intervals, effect sizes, degrees of freedom and *P* value noted<br>*Give P values as exact values whenever suitable.*
☐	☒	For Bayesian analysis, information on the choice of priors and Markov chain Monte Carlo settings
☐	☒	For hierarchical and complex designs, identification of the appropriate level for tests and full reporting of outcomes
☐	☒	Estimates of effect sizes (e.g. Cohen's *d*, Pearson's *r*), indicating how they were calculated

Our web collection on statistics for biologists contains articles on many of the points above.

Software and code

Policy information about availability of computer code

Data collection	Matlab R2016b and Psychtoolbox 3.0.12
Data analysis	R 3.6.3, RStudio, JAGS 4.3.0 and the brms package. Essential code has been made available at the Open Science Framework: https://osf.io/n7cus/?view_only=da41dfe1bf7149fab0d4c1f4690644cd

For manuscripts utilizing custom algorithms or software that are central to the research but not yet described in published literature, software must be made available to editors and reviewers. We strongly encourage code deposition in a community repository (e.g. GitHub). See the Nature Portfolio guidelines for submitting code & software for further information.

Data

Policy information about availability of data

All manuscripts must include a data availability statement. This statement should provide the following information, where applicable:

- Accession codes, unique identifiers, or web links for publicly available datasets
- A description of any restrictions on data availability
- For clinical datasets or third party data, please ensure that the statement adheres to our policy

The Data generated have been made available at the Open Science Framework.
https://osf.io/n7cus/?view_only=da41dfe1bf7149fab0d4c1f4690644cd

Field-specific reporting

Please select the one below that is the best fit for your research. If you are not sure, read the appropriate sections before making your selection.

☐ Life sciences ☒ Behavioural & social sciences ☐ Ecological, evolutionary & environmental sciences

For a reference copy of the document with all sections, see nature.com/documents/nr-reporting-summary-flat.pdf

Behavioural & social sciences study design

All studies must disclose on these points even when the disclosure is negative.

Study description	Quantitative experimental
Research sample	The study tested healthy young volunteers (n=35, age 19-37 years) recruited through the UZH and ETH web page available for recruitment of participants. The sample is representative of young healthy individuals studying at the ETH Zurich and the UZH. Due to bad eye-tracking recording quality two subjects have been excluded from analysis. In addition to the data collected in our lab, we also analyze the choice data from Folke et. al. This dataset consists of n=28 healthy young volunteers.
Sampling strategy	Sample size was determined based on previous studies using similar stimuli and tasks (Efficient coding of subjectivevalue. Nature Neuroscience, Polania, 2019; Neural Oscillations and Synchronization Differentially Support Evidence Accumulation in Perceptual and Value-Based Decision Making, Neuron, Polania, 2014; The precision of value-based choices depends causally on fronto-parietal phase coupling, Nature Communications, Polania, 2015).
Data collection	The experiment was implemented in Matlab with use of Psychtoolbox. Participants eye movements were recorded throughout the choice task at 1,000 Hz with an EyeLink 1000 Plus eyetracker (SR Research). Nobody was present during the experiment except for the participant and experimenter. The researcher was blinded to the study hypothesis.
Timing	From October 2016 until May 2017
Data exclusions	Due to bad eye-tracking recording quality two subjects have been excluded from analysis, an exclusion criteria that was pre-established. Also to make sure that participants deliberated between the two alternatives, we excluded trials where participants had not fixated on every option available at least once, this way we removed 1.1% of trials. Re-analysing the data including these trials we find no major differences.
Non-participation	No participants dropped out or declined participation.
Randomization	Participants were not allocated to experimental groups.

Reporting for specific materials, systems and methods

We require information from authors about some types of materials, experimental systems and methods used in many studies. Here, indicate whether each material, system or method listed is relevant to your study. If you are not sure if a list item applies to your research, read the appropriate section before selecting a response.

Materials & experimental systems

n/a	Involved in the study	
☒	☐	Antibodies
☒	☐	Eukaryotic cell lines
☒	☐	Palaeontology and archaeology
☒	☐	Animals and other organisms
☐	☒	Human research participants
☒	☐	Clinical data
☒	☐	Dual use research of concern

Methods

n/a	Involved in the study	
☒	☐	ChIP-seq
☒	☐	Flow cytometry
☒	☐	MRI-based neuroimaging

Human research participants

Policy information about studies involving human research participants

Population characteristics	None of the participants suffered from any neurological or psychological disorder or took medication that interfered with participation in our study. Participants where between 19 and 37 years old and from both sexes.
Recruitment	Healthy young volunteers were recruited through the UZH and ETH web page available for recruitment of participants. We are not aware of any self-selection biases that may impact the results.
Ethics oversight	The experiments conformed to the Declaration of Helsinki and the experimental protocol was approved by the Ethics Committee of the Canton of Zurich.

Note that full information on the approval of the study protocol must also be provided in the manuscript.
